# Computerized Motion Sensitivity Screening Tests in a Multicountry Rural Onchocercal Community Survey in Africa

**DOI:** 10.4103/0974-9233.71597

**Published:** 2010

**Authors:** O. E. Babalola, R. E. Umeh, A. O. Mahmoud

**Affiliations:** Rachel Eye Center, P.O. Box 4108, Garki Abuja, Nigeria; 1 Department of Ophthalmology, University of Nigeria, Enugu, Nigeria; 2 Department of Ophthalmology, University of Ilorin Teaching Hospital, Ilorin, Nigeria

**Keywords:** Blindness, Motion Sensitivity, Onchocerciasis, Rural Africa

## Abstract

**Purpose:**

To determine whether the Wu–Jones Motion Sensitivity Screening Test (MSST) accurately reflects the burden of optic nerve disease in several onchoendemic communities in Africa.

**Materials and Methods:**

The MSST was used to evaluate subjects in the communities of Raja in Sudan, Bushenyi in Uganda, Morogoro in Tanzania, and Ikon, Olomboro, and Gembu in Nigeria. Motion sensitivity was expressed as a percentage of motion detected in the individual eye, and this was averaged for the community. A perfectly normal eye would detect all motion and score 100%.

**Results:**

In this study, 3858 eyes of 2072 subjects were tested. The test was completed in 76% of respondents. Acceptability was high. Average test time was 120.4 s. The overall mean motion sensitivity of all eyes tested was 88.49%, ±17.49. Using a cutoff level of 50%, 6.4% of all subjects tested were subnormal. The highest proportion of subnormals recorded was in Morogoro at 12.7%. Severe defects in a community best correlated with optic nerve disease prevalence, while the proportion of the defect from a higher cutoff level best correlated with overall ocular morbidity. A repeat examination in the next 5 years following ivermectin treatment will show the influence, if any, on community-wide MSST performance.

**Conclusion:**

A wide range in community scores reflected disease diversity. The MSST appears to be a useful test in community-wide screening and diagnosis as it reflects the general level of ocular pathology and specifically, optic nerve disease.

## INTRODUCTION

Studies on onchocerciasis have shown that major pathways to blindness from onchocerciasis include chronic corneal disease, such as sclerosing keratitis common in the Sudan savanna regions and chorioretinal disease such as optic nerve disease and choroidoretinitis common in the rain forest^1^ and forest-savanna mosaic.^2^ Lesions of the posterior segment manifest clinically only after considerable damage to the retinal nerve fibers. By this time, damage to the optic nerve is irreversible.

Measurement of visual fields using conventional perimeters such as the Freidman field analyser, is too cumbersome for field studies and lack the sensitivity to detect early damage to the retinal nerve fibers. The computerized Motion Sensitivity Screening Test (MSST), which measures visual field using a laptop computer, has been used in onchocerciasis endemic communities in the Nigerian savanna and forest-savanna mosaic zones as well as in western countries.^3^–^6^ In these studies, adequate reliability and reproducibility were reported. The results from these previous studies indicated MSST minimized operator influence and detected early changes to optic nerve function due to either glaucoma or onchocerciasis (Personal Communication Wu, January 2006). The portability of the MSST makes it conducive to field studies and community-wide screenings compare to conventional perimeters. The MSST was been recently used in a number of onchoendemic communities as part of an international multisite impact assessment study of community-directed treatment with ivermectin (CDTI) commissioned by the African programme for onchocerciasis control (APOC). These studies will provide baseline data on the long-term impact of CDTI.

The objective of the current ophthalmic aspect of this multidisciplinary study was to determine the change in prevalence of onchocercal eye lesions, visual function defects, and blindness using standard clinical methods of eye examination and the Wu–Jones computerized visual function test (CVFT), a component of which is the MSST. The hypothesis being tested is that CDTI will prevent or delay the progression of onchocercal eye lesions and blindness and may cause regression of the lesions in the early stages of the disease.

## MATERIALS AND METHODS

### Study sites

The MSST as described by Wu *et al*.^4^ was applied to 14 onchoendemic communities. Six communities were in Anglophone countries, whereas eight were in Francophone countries. This study focuses on the six Anglophone communities. Three of these communities were in Nigeria consisting of the Ikom Local Government Area (LGA) (Etikpe, Nkarasi 11 and Nkonfap villages) in Cross River State Sardauna and Gashaka LGAs (Lemme Furwa, Lainga, Karamti and Jamtari villages) in Taraba State, and Olamaboro LGA (Ogbodo, Ubalu, Inelle, and Unyi Erogu villages) in Kogi State. The other three communities were Raja (Hai mater, Hai Manga, Wau Jadid, Hai Nasir, and Mayonga villages) in Sudan, Bushenyi in Uganda, and Morogorro in Tanzania. For political reasons, we were unable include analysis of the francophone countries in this paper.

### Community selection and sampling method

Clusters of communities were selected based on meso- or hyper-endemicity of onchocerciasis as defined by the results of Rapid Epidemiological Mapping of Onchocerciasis (World Health Organization) with specific attention to communities which had not received ivermectin (virgin communities) and communities with less than 25% ivermectin coverage of the local population.

At each site, 1500 individuals were randomly selected from a sampling frame of randomly selected households in these communities. Of this sample, 750 individuals aged 5 years and older were randomly selected for skin examination. Some individuals aged 10 years and above were selected from this list by random sampling for eye examination and the Wu–Jones MSST.

The Wu–Jones MSST has been described elsewhere.^4^ Briefly, six points within the central field of vision were repeatedly tested at 1/3 meter from the screen of a laptop computer in a room darkened either with black curtains or windows shut to filter out daylight. Very low levels of light were used in the examining room to enable the respondents and the computer operator to observe the computer screen, record the results, and allow the subjects and examiners to move about safely. With the subject seated comfortably in front of the computer, the test was explained in the local language. The subjects head was positioned on the chin rest in front of the computer. The computer then selected the eye was to be tested first (usually, the right eye). A series of vertical, oscillating illuminated white targets appeared on the screen arranged in a 6 × 8 array. Six of these points which coincide with critical points in the central field of vision were tested by oscillatory movement of the corresponding bars. Each point was tested six times. The patient pressed the space bar on the computer, or clicked on a mouse when motion was detected. This response was recorded automatically in the computer system. Detection of motion between 0–2 times out of six was categorized as a severe defect, 3–4 as a moderate defect, and 5–6 as normal. These scores were then aggregated for each eye to give an overall impression of the visual field. Twelve or less was considered a severe field defect, 24 or less was considered a moderate defect, and scores above 24 were classified as normal.

Motion sensitivity was computed as a percentage of the maximum score of 36. An eye detecting all motions is 100% sensitive. The average sensitivity of a community can thus be computed. Local nurses or assistants were trained to perform the test to mainly illiterate communities. The findings were entered on each subject’s record form as backup data. Analysis was carried out at APOC headquarters in Ouagadougou using SPSS software package. Pearson correlation coefficient (*r*) was used to determine the association between variables. *P* < 0.05 was considered statistically significant.

## RESULTS

For the six sites under review, the MSST was performed on a total of 3858 eyes comprised of 2072 right eyes and 1786 left eyes. Mean reliability of the test was 69.8 ± 34.6%. The duration of the test varied from 36 to 1158 s with a mean of 120.4 ± 66.7 s. The test was generally acceptable to the subjects, and it was possible to complete it in 2072 subjects. The test could not be performed or could not be completed in 24% of all cases.

**Figure 1:**
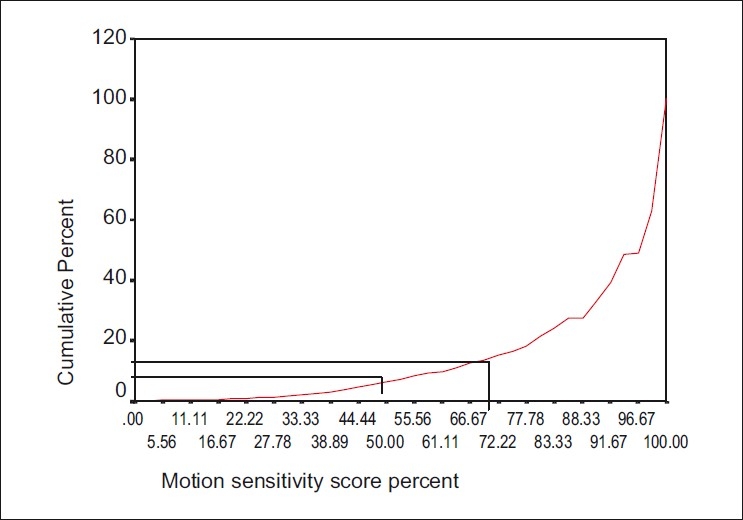
Composite MSST scores in 3858 eyes of residents of six onchoendemic communitie

The overall mean motion sensitivity for all eyes tested was 88.49 ± 17.5%. Of the total number of eyes tested, 3375 (87.5%) were normal, 405 (10.5%) had moderate field loss, and 78(2.0%) had severe loss. Using a motion sensitivity cutoff level of 50%, 6.4% of eyes were subnormal. An increase in the cutoff level to 70%, resulted in 13.3% subnormal eyes. Figure 1 plots the cumulative frequencies of MSST scores of the study cohort and the cutoff levels. The lack of smoothness in the curves in Figure 1 makes it appear that at least three curves have been superimposed. This reflects the different curves in the various communities combined. There was a weak but statistically significant negative correlation between MSST scores and age (*r* = −0.130, *P* < 0.01).

### Results by site

**Figure 2:**
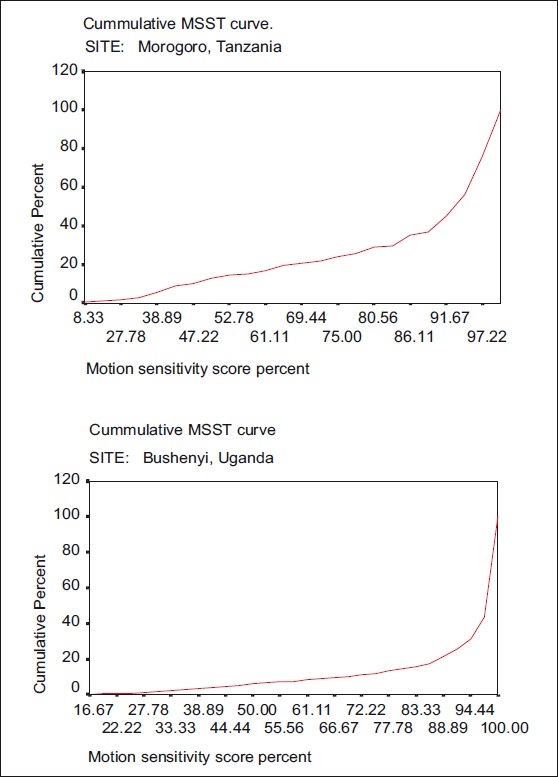
Comparing cumulative scores in two places. Note the relative “shift” to the right of the curve for Bushenyi, which is indicative of a lower rate of subnormal MSST in the Bushenyi population, and a reflection of the lower overall prevalence of ocular morbidity

Table 1 presents the prevalence of normal, moderate, and severe field loss in the various sites as well as the mean MSST score per site. The highest mean MSST score was recorded in Raja, Sudan at 91.9% and the lowest in Kogi, Nigeria at 83.4%. Table 1also presents the proportion of subnormal MSST scores per site using the 50% cutoff level. The highest proportion was recorded in subjects from Morogoro, Tanzania at 12.7% whereas the lowest was recorded in Ikom, Cross River site, Nigeria at 3.9%. Figure 2 plots comparison of cumulative scores at two sites.

**Table 1:** Results from Wu–Jones motion sensitivity screening test in various oncho-endemic sites in Africa

Site	Number of examined eyes (MSST)	Moderate field loss	Severe field loss	Any field loss	Subnormal MSST (≤50%)	Mean MSST score (%)
Raja (Sudan)	719	31 (4.3)	20 (2.8)	51 (7.1)	4.5	91.9
Kogi (Nigeria)	551	111 (20.1)	14 (2.5)	125 (22.6)	10.5	83.4
Taraba (Nigeria)	850	112 (13.2)	18 (2.1)	130 (15.3)	6.6	86.1
Ikom (Nigeria)	894	71 (7.9)	9 (1.0)	80 (8.9)	3.9	89.4
Bushenyi (Uganda)	679	51 (7.5)	14 (2.1)	65 (9.6)	6.3	91.8
Morogoro (Tanzania)	165	29 (17.6)	3 (1.8)	32 (19.4)	12.7	84.7

MSST: Motion sensitivity screening test, Figures in parentheses are in percentage

### Correlation with ocular pathology

The prevalence of certain selected ocular pathology indicators by site is presented in Table 2. The strongest correlations were observed between the proportion of severe field loss and the prevalence of optic atrophy yet were not statistically significant (*r* = 0.54, *P* = 0.27). Subnormal MSST scores at the 50% cutoff level correlated best with the combined prevalence of blindness and visual impairment at *r* = 0.66, *P* = 0.155.

**Table 2:** Selected ocular pathology prevalences in the onchoendemic sites

Site	Optic atrophy (%)	Glaucoma (%)	Optic atrophy + glaucoma (%)	Chorioretinits (%)	Sclerosing keratitis (%)	Cataract (%)	Blindness prevalence (%)	Visual impairment (%)	Blindness+ visual impairment (%)
Raja (Sudan)	21.5	13.8	35.3	16.7	12.2	13.1%	10.3	7.9%	18.2
Kogi (Nigeria)	5.3	2.3	7.6	4.3	0.0	9.8	3.3	13.3	16.6
Taraba (Nigeria)	7.3	5.2	12.5	3.1	6.5	9.1	3.2	6.3	9.5
Ikom (Nigeria)	7.3	12.4	19.7	35.8	6.3	7.5	2.8	10.3	13.1
Bushenyi (Uganda)	12.4	1.4	13.8	3.3	3.8	6.3	1.9	3.9	5.8
Morogoro	4.8	4.2	9.0	2.6	4.6	27.4	15.3	12.7	30

## DISCUSSION

Wu *et al*.^4^ had postulated that the MSST is a useful mass screening device for epidemiological studies of diseases such as onchocerciasis and glaucoma that commonly produce visual field loss while preserving macular function. This study documents what may be the first time since Wu *et al*.^4^ publication that MSST has been used on such a large scale (e.g., cohort size and locales). A previous study was conducted by Umeh *et al*. on approximately 100 subjects in South Eastern Nigeria.^5^ In this study, 24% of the subjects were unable to complete the test for various reasons, including low vision (less than count fingers) and which resulted in poor fixation on the central target which is fundamental for the MSST. This was particularly the case in Raja, where the prevalence of visual loss was high with a considerable number of the subjects suffering from optic nerve disease yet only a small percentage of subnormal MSST was recorded.

We found high acceptability of the test, similar to the observations of Quigley *et al*.^6^ However, alternatives to the MSST exist. Field tests using Friedman semi-automated perimetry have been previously published.^1^,^7^ In a randomized, controlled trial of annual ivermectin for onchocerciasis, Murdoch *et al*.^8^ have shown that it was possible to obtain reliable and reproducible perimetric data with the Friedman perimeter. However, Freidman perimeter is large, and relatively cumbersome compared to a laptop. Additionally, the Friedman perimeter requires an external power source to function whereas the MSST requires a fully charged computer battery that can operate for up to 2 h without an external power source. Another potential advantage of the MSST is the ability to detect early damage to large ganglion retinal fibers which may be the fibers affected in onchocerciasis (unpublished report by Wu *et al*.).

In this study, most subjects were able to understand the test once it was explained to them in their own language. The completion time for the test excluding the time for explanation, averaged about 2 min. In the field, we saved significant time by explaining the test to a group of subjects at one time using local subjects who had successfully completed the test. Mean reliability was almost 70% which can be considered more than adequate for the largely semi-illiterate and illiterate populations studied.

Using a cutoff level of 50% or less, 6.4% of the combined population was found to have subnormal MSST scores. This subnormal rate varied from 3.9% in Ikom to 12.7% in Morogoro. Wu *et al*.^4^ had found that 15% of the onchoendemic population in Kaduna, Nigeria had subnormal motion sensitivity loss, whereas only 5% were subnormal in the nonendemic Nigerian areas. Extending the cutoff level to 70% increased the subnormal rate to 13.3% which is substantially lower compared to the 25% reported by Wu *et al*. in a Nigerian onchoendemic population.^4^ This may suggest that the overall burden of optic nerve disease and other factors that may contribute to motion sensitivity reduction in the endemic sites we study is lower than the sites studied by Wu *et al*.^4^ Inter-observer variations may also be a factor although this was not a major issue according to Wu *et al*. with limits of agreement ranging from −0.198 to 0.18.

One drawback in our testing method was the difficulty in standardizing the ambient lighting (stray light). A background luminance of 200 lux has been suggested. One method may involve the equipping study personnel with small handheld light meters to measure room light conditions to select testing sites with the light background illumination. Standardization of background illumination in the field may significantly affect the outcome of the MSST and allow easier comparison across test sites.

The presence of “any field loss” in our population ranged from 7.1% in Raja to 19.2% in Morogoro. We found a weak but statistically significant negative correlation between MSST scores and age (*r* = −0.130, *P* < 0.01) which is lower than the −0.21 (*P* < 0.01) reported by Wu *et al*. in onchoendemic Nigerian populations. Our results indicate that there was a general trend in the reduction of MSST scores with age, although there may be other associated confounding variables such as associated ocular pathology.

There was a wide variation in MSST scores across the sites, as indicated in the results. It would be tempting to conclude that this was mainly due to variation in the prevalence of optic nerve disease. Correlations with MSST scores have to be interpreted with caution due to the small sample size of the six sites in this study. Confounding factors such as variable testing conditions at the sites and inter-observer variation may affect the data. Due to these factors, it is difficult to draw conclusions on the correlation between overall community MSST performance and the prevalence of optic nerve disease.

With the above caveats in mind, the strongest correlation (*r* = 0.54, *P* = 0.27) was observed between the prevalence of optic atrophy and severe field loss, which in our context is a field ≤33% of normal (12/36). Subnormal MSST with a 50% cutoff level correlated best with combined prevalence of blindness and visual impairment at (*r* = 0.66, *P* = 0.155). These trends are illustrated by two extreme examples of Morogoro and Bushenyi. Morogoro had the highest level of blindness and visual impairment at 30%, yet there was a relatively low prevalence of optic atrophy (4.8%), because most of the visual problems were attributable to uveitis and secondary cataracts, not pathology affecting the optic nerve.^8^ Consequently, the proportion of severe field loss, which reflects the prevalence of optic atrophy, was also low at 1.8%, yet the proportion of subnormal MSST, which reflects the overall blindness and visual impairment status, was the highest in the series at 12.7%. The prevalence of blindness and visual impairment reported here reflect the overall disease burden from diverse causes in the community. Alternately, Bushenyi had a low burden of blindness and visual impairment at 5.8% but a relatively high prevalence of optic atrophy at 12.4%. This was reflected in the relatively higher prevalence of severe field loss (2.1%), but a lower prevalence of subnormal MSST at 6.3%. To further illustrate these points, the cumulative curve for Morogoro is closer to what is expected in an endemic onchocercal community with a relative “shift” of the curve to the left, while that of Bushenyi approximates the situation in a nonendemic community. If CDTI should prove effective over time, a shift to the right is expected in cumulative MSST curves in the communities.

These results suggest that when applied in a cross-sectional manner, the proportion of severe field defects in a community are a more reliable predictor of the prevalence of optic nerve disease, while the 50% cutoff level is more indicative of overall ocular disease burden. Hopefully, as results from more sites become available, it will be possible to make more conclusive statements. What is certain, however, is that comparisons over time within communities (i.e., repetition of the MSST within the same community) should be a fairly straightforward and reliable method to assess burden of ocular disease burden. Typical patterns in the cumulative frequency curves have been previously documented by Wu *et al*.^3^,^4^ A “shift” to the right of the cumulative frequency curve is indicative of a tendency to normality. For example, the relative “shift” to the right of the curve for Bushenyi, is indicative of a lower rate of subnormal MSST in the Bushenyi population, and a reflection of the lower overall prevalence of ocular morbidity.

Of interest will be the change (if any) in the cumulative curves after 5 years of CDTI. The data at 5 years will also determine whether MSST can demonstrate changes in visual function and ocular morbidity over time, brought about by ivermectin administration.

In conclusion, the Wu–Jones MSST was applied as a cross-sectional instrument in six Anglophone onchoendemic communities. A wide range in overall community scores was noticed, probably reflective of disease diversity within the various communities studied. We hope to repeat the tests in the next 5 years following ivermectin treatment, when the influence, if any, on community-wide MSST performance will become more apparent.
